# A Prospective Observational Study on Serum Lactate Dehydrogenase and Uric Acid Levels in Hypertensive Disorders of Pregnancy and Prediction of Fetomaternal Outcome

**DOI:** 10.7759/cureus.104619

**Published:** 2026-03-03

**Authors:** Kavya B Naganagoudar, Subhashchandra R Mudanur, Rajasri G Yaliwal, Shreedevi Kori, Laxmi Sangolli

**Affiliations:** 1 Obstetrics and Gynecology, Shri BM Patil Medical College Hospital and Research Centre, BLDE (Deemed to be University), Vijayapura, IND

**Keywords:** fetal outcome, hypertensive disorders of pregnancy, lactate dehydrogenase, maternal outcome, uric acid

## Abstract

Introduction

Hypertensive disorders of pregnancy are a major cause of maternal and perinatal morbidity and mortality, particularly in low- and middle-income countries. Early identification of women at risk using simple biochemical markers may aid in timely intervention and improved outcomes. Serum lactate dehydrogenase (LDH) and uric acid have been proposed as indicators of disease severity in hypertensive pregnancies. The study aimed to evaluate the association of serum LDH and uric acid levels with the severity of hypertensive disorders of pregnancy and their relationship with maternal and fetal outcomes in women with hypertensive disorders of pregnancy.

Materials and methods

This prospective observational study was conducted in a tertiary care teaching hospital from March 2024 to December 2025. A total of 165 pregnant women with hypertensive disorders of pregnancy and gestational age ≥20 weeks were enrolled. Serum LDH and uric acid levels were measured antenatally and after delivery using standard laboratory methods. Normal values were defined as LDH <300 IU/L and uric acid <6 mg/dL. Maternal outcomes included mode of delivery and complications such as hemolysis, elevated liver enzymes, low platelet count (HELLP) syndrome, disseminated intravascular coagulation, and postpartum hemorrhage. Fetal outcomes assessed included birth weight, preterm delivery, appearance, pulse, grimace, activity, and respiration (APGAR) score, need for resuscitation, and perinatal mortality.

Results

The majority of women were aged 21-30 years, and primigravidae constituted the largest group. Elevated serum LDH and uric acid levels were observed in 85.5% (n=141) and 65.5% (n=108) of cases, respectively. Both biomarkers showed a significant progressive rise with increasing severity of hypertensive disorders. Elevated serum LDH levels were significantly associated with adverse fetal outcomes, including low birth weight, need for resuscitation, neonatal death, and preterm birth. Similarly, raised serum uric acid levels showed significant associations with need for resuscitation, neonatal death, and preterm birth. No statistically significant association was observed between elevated biomarker levels and maternal complications.

Conclusion

Serum LDH and uric acid are valuable, cost-effective biochemical markers that reflect disease severity in hypertensive disorders of pregnancy and can aid in predicting adverse maternal and fetal outcomes. However, their association with maternal complications was not statistically significant in this study.

## Introduction

Hypertensive disorders of pregnancy (HDP) remain a major contributor to maternal and perinatal morbidity and mortality worldwide, particularly in low- and middle-income countries [1,2]. Affecting approximately 3-5% of all pregnancies, these disorders account for a substantial proportion of preventable adverse outcomes [3]. HDP represents a heterogeneous group of conditions that include gestational hypertension, pre-eclampsia, eclampsia, and chronic hypertension, of which pre-eclampsia and eclampsia are associated with the most severe maternal and fetal complications due to their multisystem involvement [4,5].

Early identification of women at risk is crucial for preventing disease progression and improving fetomaternal outcomes [6]. Increasing evidence suggests that abnormal placentation, uteroplacental hypoperfusion, endothelial dysfunction, oxidative stress, and systemic inflammation play central roles in the pathogenesis of HDP [7]. Consequently, there has been growing interest in identifying reliable, easily measurable biochemical markers that reflect these underlying processes and can aid in early risk stratification and assessment of disease severity [8].

Serum uric acid has emerged as a potential marker of disease severity in hypertensive pregnancies [9]. Elevated levels may be detected early in gestation and are thought to result from impaired renal clearance, placental ischemia, and increased oxidative stress [10]. Although hyperuricemia is not universally present in all cases of pre-eclampsia, its elevation has been consistently associated with worsening maternal condition and adverse fetal outcomes, suggesting both clinical and prognostic relevance [11].

Serum lactate dehydrogenase (LDH), an intracellular enzyme released during cellular injury and hypoxia, has also been recognized as an important marker of disease severity in HDP [12,13]. Elevated LDH levels reflect widespread endothelial damage, hemolysis, and tissue hypoxia, which are characteristic of severe pre-eclampsia and eclampsia [14]. Increased LDH activity has been linked to serious complications such as HELLP syndrome, placental abruption, intrauterine growth restriction, and poor neonatal outcomes [15]. Given the wide availability, affordability, and reproducibility of serum LDH and uric acid assays, their routine assessment may provide valuable insight into disease severity and help guide clinical management.

The present study aimed to evaluate the association of serum LDH and uric acid levels with the severity of hypertensive disorders of pregnancy and their relationship with maternal and fetal outcomes.

## Materials and methods

Study design and setting

This prospective observational study was conducted in the Department of Obstetrics and Gynecology at BLDE (Deemed to be University), Shri B. M. Patil Medical College Hospital and Research Centre, Vijayapur, a tertiary care teaching hospital. The study period extended from March 2024 to December 2025. Ethical approval was obtained from the Institutional Ethics Committee (IEC No. BLDE(DU)/IEC-SBMPMC/060/2023-24).

Study population

Pregnant women diagnosed with hypertensive disorders of pregnancy who attended the antenatal outpatient department or were admitted to the inpatient wards were screened for eligibility. A total of 165 women fulfilling the inclusion criteria were consecutively enrolled after obtaining written informed consent.

Hypertensive disorders were classified according to the American College of Obstetricians and Gynecologists (ACOG) criteria. Gestational hypertension was defined as blood pressure ≥140/90 mmHg after 20 weeks of gestation without proteinuria. Preeclampsia was defined as hypertension with proteinuria (≥300 mg/24 hours) or evidence of end-organ dysfunction. Severe preeclampsia was defined by blood pressure ≥160/110 mmHg or the presence of severe features. Eclampsia was defined as the occurrence of generalized tonic-clonic seizures in a woman with preeclampsia [4].

Inclusion and exclusion criteria

Pregnant women with a singleton pregnancy of ≥20 weeks' gestation who were clinically diagnosed with hypertensive disorders of pregnancy and provided written informed consent were included in the study. Women with pre-existing cardiac, renal, or liver disease; known hyperuricemia or gout; deranged renal or liver function tests due to any medical or surgical illness; and those who declined participation were excluded from the study.

Sample size estimation

Sample size was calculated based on a previous study by Moharana et al. [16], which reported a 12.2% prevalence of serum uric acid levels >8 mg/dL associated with NICU admission. Using a confidence level of 95% and a margin of error of 5%, the sample size was estimated using the formula: n=z^2^×p×(1−p)​/d^2^, where z=1.96, p=0.122, and d=0.05. The calculated sample size was 165 participants.

Data collection

After enrolment, detailed demographic, medical, and obstetric histories were recorded using a structured proforma. General physical and obstetric examinations were performed for all participants. Venous blood samples were collected at the time of clinical diagnosis or admission, prior to definitive management, for estimation of serum LDH and serum uric acid levels. Serum LDH was measured using the kinetic method, and serum uric acid was analyzed using the uricase method. Biochemical assessments were performed antenatally and repeated after delivery. Normal serum LDH was defined as <300 IU/L, and normal serum uric acid as <6 mg/dL [16].

Outcome measures

Maternal outcomes included progression of hypertensive disease and development of complications such as eclampsia, hemolysis, elevated liver enzymes, low platelet count (HELLP) syndrome, disseminated intravascular coagulation, placental abruption, postpartum hemorrhage, and maternal mortality, along with mode of delivery. Fetal outcomes assessed included birth weight, preterm delivery, appearance, pulse, grimace, activity, and respiration (APGAR) score at five minutes, need for neonatal resuscitation, intrauterine death, neonatal death, and NICU admission.

Statistical analysis

Data were entered into Microsoft Excel (Microsoft Corp., Redmond, US) and analyzed using SPSS (version 26; IBM SPSS Statistics for Windows, Armonk, US). Continuous variables were expressed as mean±standard deviation and compared using one-way ANOVA. Categorical variables were summarized as frequencies and percentages and compared using the chi-square test or Fisher's exact test as appropriate. A p-value <0.05 was considered statistically significant.

## Results

The study population predominantly comprised women aged 21-30 years, with primigravida constituting nearly two-thirds of patients. Preterm delivery was more frequent than term delivery, and lower segment cesarean section slightly exceeded vaginal delivery as the mode of birth, reflecting the high-risk nature of the cohort (Table 1).

**Table 1:** Baseline maternal and obstetric characteristics LSCS - lower segment cesarean section

Variable	Category	n (%)
Age (years)	18-20	18 (10.9)
	21-30	125 (75.8)
	31-40	20 (12.1)
	>40	2 (1.2)
Gravida	Primigravida	105 (63.6)
	Multigravida	60 (36.4)
Time of delivery	Preterm	90 (54.5)
	Term	52 (31.5)
	Post-term	23 (13.9)
Mode of delivery	Vaginal	76 (46.1)
	Assisted vaginal	10 (6.1)
	LSCS	79 (47.8)

Pre-eclampsia emerged as the dominant hypertensive disorder, with mild and severe forms together accounting for more than half of the cases. Eclampsia contributed a substantial proportion, while gestational hypertension was comparatively less common (Table 2).

**Table 2:** Distribution of hypertensive disorders of pregnancy

Hypertensive disorder	n (%)
Gestational hypertension	30 (18.2)
Mild pre-eclampsia	51 (30.9)
Severe pre-eclampsia	48 (29.1)
Eclampsia	36 (21.8)

Biochemical evaluation revealed that elevated serum LDH was highly prevalent, affecting more than four-fifths of participants, whereas abnormal serum uric acid levels were observed in approximately two-thirds of cases, indicating widespread biochemical derangement in hypertensive pregnancies (Table 3).

**Table 3:** Distribution of biochemical parameters Normal values were defined as LDH <300 IU/L and serum uric acid <6 mg/dL. LDH - lactate dehydrogenase

Parameter	Normal, n (%)	Abnormal, n (%)
Serum LDH	24 (14.5)	141 (85.5)
Serum uric acid	57 (34.5)	108 (65.5)

Both serum LDH and uric acid levels demonstrated a clear and progressive rise with increasing severity of hypertensive disorders, with the lowest mean values observed in gestational hypertension and the highest levels in eclampsia. This graded increase was statistically significant for both biomarkers, suggesting a strong association with disease severity (Table 4).

**Table 4:** Hypertensive disorders and mean serum LDH and uric acid levels Comparison of mean serum LDH and serum uric acid levels across different hypertensive disorders of pregnancy. Statistical comparison between groups was performed using one-way ANOVA. F-values and corresponding p-values indicate a significant progressive rise in both biomarkers with increasing disease severity. LDH - lactate dehydrogenase

Disorder	n	Mean LDH (U/L)	Mean uric acid (mg/dL)
Gestational hypertension	30	223.3±23.2	4.3±0.7
Mild pre-eclampsia	51	431.5±67.0	6.2±0.6
Severe pre-eclampsia	48	768.3±93.9	7.5±0.9
Eclampsia	36	1020.3±138.4	8.5±0.9
F-value		215.653	184.291
p-value		<0.0001	<0.0001

Abnormal serum LDH levels were frequently noted across all maternal complications, including postpartum hemorrhage, disseminated intravascular coagulation (DIC), HELLP syndrome, and maternal death. However, none of these associations reached statistical significance, indicating that elevated LDH was common but not significantly associated with specific maternal complications in this cohort (Table 5).

**Table 5:** Maternal complications according to serum LDH levels Statistical significance was tested using chi-square or Fisher's exact test, as appropriate. No maternal complication showed a statistically significant association with elevated serum LDH. LDH - lactate dehydrogenase; PPH - postpartum hemorrhage; DIC - disseminated intravascular coagulation; HELLP - hemolysis, elevated liver enzymes, low platelet count

Maternal complication	Total, n	Normal LDH, n (%)	Abnormal LDH, n (%)	Chi-square value	p-value
PPH	24	3 (12.5)	21 (87.5)	0.094	0.758
Abruption placenta	4	0 (0.0)	4 (100)	0.697	0.403
DIC	22	3 (13.6)	19 (86.4)	0.15	0.70
HELLP syndrome	20	5 (25.0)	15 (75.0)	1.12	0.29
Wound discharge	3	0 (0.0)	3 (100)	0.520	0.471
Maternal death	2	0 (0.0)	2 (100)	0.344	0.557

Also, elevated serum uric acid did not show a significant association with maternal complications, including postpartum hemorrhage, DIC, HELLP syndrome, and maternal death (Table 6).

**Table 6:** Maternal complications according to serum uric acid levels Chi-square or Fisher's exact test was applied as appropriate. No maternal complication demonstrated a statistically significant association with elevated serum uric acid levels. PPH - postpartum hemorrhage; DIC - disseminated intravascular coagulation; HELLP - hemolysis, elevated liver enzymes, low platelet count

Maternal complication	Total, n	Normal uric acid, n (%)	Abnormal uric acid, n (%)	Chi-square value	p-value
PPH	24	8 (33.3)	16 (66.7)	0.01	0.89
Abruption placenta	4	1 (25.0)	3 (75.0)	0.16	0.68
DIC	22	14 (63.6)	8 (36.4)	2.31	0.12
HELLP syndrome	20	3 (15.0)	17 (85.0)	0.07	0.78
Wound discharge	3	1 (33.3)	2 (66.7)	0.47	0.49
Maternal death	2	1 (50.0)	1 (50.0)	1.3	0.23

Adverse fetal outcomes were commonly associated with abnormal serum LDH levels, with statistically significant associations observed for low birth weight, need for neonatal resuscitation, neonatal death, and preterm birth. These findings suggest that elevated LDH is significantly associated with adverse fetal outcomes in hypertensive pregnancies (Table 7).

**Table 7:** Fetal outcomes according to serum LDH levels Statistical analysis was performed using chi-square or Fisher's exact test, as appropriate. Elevated LDH levels showed significant associations with low birth weight, need for neonatal resuscitation, neonatal death, and preterm birth. LDH - lactate dehydrogenase; APGAR - appearance, pulse, grimace, activity, respiration score; IUD - intrauterine death

Fetal outcome	Total, n	Normal LDH, n (%)	Abnormal LDH, n (%)	Chi-square value	p-value
Low birth weight	50	4 (8.0)	46 (92.0)	3.89	0.048
Five-minutes APGAR <7	11	2 (18.2)	9 (81.8)	0.45	0.50
Need for resuscitation	9	0 (0.0)	9 (100)	42.32	<0.0001
IUD	2	1 (50.0)	1 (50.0)	2.04	0.15
Neonatal death	8	5 (62.5)	3 (37.5)	10.67	0.001
Preterm birth	32	10 (31.2)	22 (68.8)	8.91	0.002

Abnormal serum uric acid levels were also frequently present in adverse fetal outcomes, with significant associations noted for need for resuscitation, neonatal death, and preterm birth, while correlations with low birth weight and low APGAR scores did not reach statistical significance (Table 8).

**Table 8:** Fetal outcomes according to serum uric acid levels Chi-square or Fisher's exact test was applied as appropriate. Significant associations were observed for neonatal resuscitation, neonatal death, and preterm birth, while other outcomes were not statistically significant. APGAR - appearance, pulse, grimace, activity, respiration score; IUD - intrauterine death

Fetal outcome	Total, n	Normal uric acid, n (%)	Abnormal uric acid, n (%)	Chi-square value	p-value
Low birth weight	50	12 (24.0)	38 (76.0)	2.87	0.09
Five-minutes APGAR <7	11	1 (9.1)	10 (90.9)	2.31	0.12
Need for resuscitation	9	8 (88.9)	1 (11.1)	12.43	0.0004
IUD	2	0 (0.0)	2 (100)	0.54	0.3012
Neonatal death	8	0 (0.0)	8 (100)	0.09	0.0351
Preterm birth	32	4 (12.5)	28 (87.5)	8.53	0.003

## Discussion

The present study demonstrates that hypertensive disorders of pregnancy predominantly affect women in their early reproductive years, with the majority of cases occurring between 21 and 30 years of age. This age distribution is consistent with previous reports by Moharana et al., Awoyesuku et al., Preet et al., and Saha et al., all of whom documented a higher prevalence of hypertensive disorders among women below 30 years of age [16-19]. The predominance of primigravidae in the present study further aligns with established epidemiological patterns reported in earlier literature, supporting the recognized vulnerability of first pregnancies to hypertensive complications [16,18].

A high rate of preterm delivery was observed in the present cohort, reflecting disease severity and the frequent need for early termination of pregnancy in women with hypertensive disorders. Similar findings have been reported by Moharana et al. and Awoyesuku et al., who documented preterm birth rates ranging from 60% to 72% among women with elevated LDH levels [16,17]. Minor variation reported by Saha et al., who observed lower preterm delivery rates, may be attributed to earlier diagnosis and timely clinical intervention [19]. These observations collectively reinforce the strong association between severe hypertensive disease, biochemical abnormalities, and medically indicated preterm birth.

In the present study, abnormal serum LDH levels were observed in the majority of women, indicating widespread cellular injury, hemolysis, and endothelial dysfunction, which are hallmark features of severe preeclampsia and eclampsia. This finding is in agreement with earlier studies reporting a high prevalence of LDH elevation in severe hypertensive disorders of pregnancy. Awoyesuku et al. reported markedly elevated LDH levels exceeding 700 IU/L in severe cases, which were associated with adverse fetal outcomes [17]. Comparable observations were reported by Moharana et al., Saha et al., and Ajila et al., who emphasized serum LDH as a reliable marker of disease severity and fetoplacental compromise [16,19,20].

Elevated serum uric acid levels were observed in nearly two-thirds of women in the present study, reflecting impaired renal function and increased oxidative stress. Similar associations between hyperuricemia and disease severity in preeclampsia have been reported by Kumar et al. and Saha et al. [9,19]. Moharana et al. also reported significantly elevated uric acid levels in severe disease, correlating with adverse fetal outcomes such as low birth weight and preterm delivery [16]. Compared with LDH, uric acid elevation was less frequent in the present study, suggesting that LDH may reflect more generalized cellular injury, whereas uric acid more specifically mirrors renal involvement and oxidative stress.

Both serum LDH and uric acid levels demonstrated a progressive rise with increasing severity of hypertensive disorders, from gestational hypertension to eclampsia, a trend consistently reported across multiple studies [16-20]. Elevated serum LDH levels were significantly associated with adverse fetal outcomes, including low birth weight, need for resuscitation, neonatal death, and preterm birth. Similarly, raised serum uric acid levels showed significant associations with need for resuscitation, neonatal death, and preterm birth. These findings suggest complementary roles for the two biomarkers in hypertensive disorders of pregnancy, with LDH serving as a sensitive indicator of fetoplacental compromise and uric acid reflecting maternal disease severity.

However, in the present study, neither serum LDH nor uric acid demonstrated a statistically significant association with maternal complications. This may be partly attributable to the relatively low frequency of certain maternal adverse events, potentially limiting statistical power to detect meaningful differences.

Strengths and limitations

The strengths of this study include its prospective design, adequate sample size, and systematic evaluation of both maternal and fetal outcomes using widely available and cost-effective biochemical markers. The use of standardized laboratory methods and clearly defined cut-off values for serum LDH and uric acid enhances the reliability and reproducibility of the findings, while inclusion of a broad spectrum of hypertensive disorders allows meaningful assessment of disease severity.

However, certain limitations merit consideration. As a single-center study conducted in a tertiary care hospital, the findings may not be fully generalizable to other healthcare settings. Long-term maternal and neonatal outcomes beyond the immediate perinatal period were not assessed, restricting evaluation of the prolonged prognostic significance of these biochemical markers. In addition, biochemical parameters were measured at a single antenatal time point, and serial measurements were not performed. Therefore, dynamic trends in LDH and uric acid levels could not be evaluated. The analysis was primarily univariate, and multivariate regression to adjust for potential confounding variables such as maternal age, parity, and gestational age at diagnosis was not performed. Hence, the independent predictive value of these biomarkers cannot be definitively established.

## Conclusions

Hypertensive disorders of pregnancy continue to be a significant cause of maternal and perinatal morbidity and mortality, particularly in resource-limited settings. The present study demonstrates that serum lactate dehydrogenase and uric acid levels increase progressively with the severity of hypertensive disease and are significantly associated with adverse fetal outcomes. Elevated serum LDH and uric acid showed stronger associations with poor fetal outcomes, suggesting their potential role as markers of fetoplacental compromise. However, no statistically significant association was observed between these biomarkers and maternal complications in this cohort. Routine assessment of these easily available biochemical markers, when interpreted in conjunction with clinical findings, may aid in early identification of high-risk pregnancies, enable timely intervention, and contribute to improved fetal and perinatal outcomes.

## References

[1] Zerihun E, Girma F, Amena N (2025). Effect of hypertensive disorders of pregnancy (HDP) on maternal and perinatal birth outcomes in Eastern Ethiopia: a prospective cohort study. BMC Pregnancy Childbirth.

[2] Muthulingam D, Murugesan A, Sankar G (2025). Outcomes of pregnancy complicated by hypertension: a study of maternal and fetal health. Indian J Obstet Gynecol Res.

[3] Khedagi AM, Bello NA (2021). Hypertensive disorders of pregnancy. Cardiol Clin.

[4] American College of Obstetricians and Gynecologists (2019). ACOG practice bulletin No. 203: chronic hypertension in pregnancy. Obstet Gynecol.

[5] Booker WA (2020). Hypertensive disorders of pregnancy. Clin Perinatol.

[6] Santos J, Schenone MH, Garovic VD (2023). Early identification of individuals at risk for hypertensive disorders of pregnancy. JAMA Netw Open.

[7] Phoswa WN, Khaliq OP (2021). The role of oxidative stress in hypertensive disorders of pregnancy (preeclampsia, gestational hypertension) and metabolic disorder of pregnancy (gestational diabetes mellitus). Oxid Med Cell Longev.

[8] Zhou C, Song C, Huang X (2021). Early prediction model of gestational hypertension by multi-biomarkers before 20 weeks gestation. Diabetes Metab Syndr Obes.

[9] Kumar N, Singh AK (2019). Maternal serum uric acid as a predictor of severity of hypertensive disorders of pregnancy: a prospective cohort study. Curr Hypertens Rev.

[10] Arias-Sánchez C, Pérez-Olmos A, Reverte V, Hernández I, Cuevas S, Llinás MT (2025). Uric acid and preeclampsia: pathophysiological interactions and the emerging role of inflammasome activation. Antioxidants.

[11] Omar Wehlie H, Fajardo Tornes Y, Businge J (2025). Association between hyperuricemia and adverse perinatal outcomes among women with preeclampsia at a tertiary hospital in Southwestern Uganda: a prospective cohort study. J Matern Fetal Neonatal Med.

[12] Teklemariam AB, Abebe EC, Agidew MM, Ayenew AA, Mengistie MA, Baye ND, Muche ZT (2024). Diagnostic performance of lactate dehydrogenase as a potential biomarker in predicting preeclampsia and associated factors. Front Med.

[13] Kumari N, Bala R, Pahwa S (2024). Lactate dehydrogenase as a biochemical marker for prediction of maternal and perinatal outcomes in hypertensive disorders in pregnancy. Indian J Obstet Gynecol Res.

[14] Raghuwanshi K, Kushram B, Dandotiya D, Petkar S, Tambade S, Gandhe M (2025). Lactate dehydrogenase (LDH) as an indicator of pre-eclampsia. Bioinformation.

[15] Nguyen KD, Tn Khuc A, A Tran V, S Nguyen H, H Nguyen P, Th Nguyen A (2025). Antepartum serum lactate dehydrogenase and adverse obstetric outcomes in preeclampsia: a systematic review of prognostic evidence from low- and middle-income countries. Cureus.

[16] Moharana JJ, Mishra R, Nayak AK (2023). A study on serum lactate dehydrogenase and uric acid in preeclampsia and eclampsia: can they predict adverse fetomaternal outcome?. Int J Appl Basic Med Res.

[17] Awoyesuku PA, Ohaka C, Altraide BO (2024). Maternal serum lactate dehydrogenase level as a predictor of adverse pregnancy outcome in women with severe preeclampsia. Int J Reprod Contracept Obstet Gynecol.

[18] Preet A, Anand AR (2023). A study on lactate dehydrogenase levels in hypertensive disorders of pregnancy and its correlation with feto-maternal outcome. Int J Reprod Contracept Obstet Gynecol.

[19] Saha M, Rohman SM, Das KK (2018). A study of serum lactate dehydrogenase and uric acid level in preeclampsia in a tertiary health care centre in North East India. J Med Sci Clin Res.

[20] Ajila D, Raja A, Ganiga P (2021). Maternal serum lactate dehydrogenase level and adverse pregnancy outcomes in women with hypertensive disorder of pregnancy at a tertiary care centre: a retrospective study. Int J Reprod Contracept Obstet Gynecol.

